# Clathrin Is Spindle-Associated but Not Essential for Mitosis

**DOI:** 10.1371/journal.pone.0003115

**Published:** 2008-09-03

**Authors:** Joana Borlido, Greg Veltri, Antony P. Jackson, Ian G. Mills

**Affiliations:** 1 Uro-Oncology Research Group, CRUK Cambridge Research Institute, Li Ka Shing Cancer Research Centre, Cambridge, United Kingdom; 2 Flow Cytometry Core, CRUK Cambridge Research Institute, Li Ka Shing Cancer Research Centre, Cambridge, United Kingdom; 3 Department of Biochemistry, University of Cambridge, Cambridge, United Kindom; Thomas Jefferson University, Kimmel Cancer Center, United States of America

## Abstract

**Background:**

Clathrin is a multimeric protein involved in vesicle coat assembly. Recently clathrin distribution was reported to change during the cell cycle and was found to associate with the mitotic spindle. Here we test whether the recruitment of clathrin to the spindle is indicative of a critical functional contribution to mitosis.

**Methodology/Principal Findings:**

Previously a chicken pre-B lymphoma cell line (DKO-R) was developed in which the endogenous clathrin heavy chain alleles were replaced with the human clathrin heavy chain under the control of a tetracycline-regulatable promoter. Receptor-mediated and fluid-phase endocytosis were significantly inhibited in this line following clathrin knockout, and we used this to explore the significance of clathrin heavy chain expression for cell cycle progression. We confirmed using confocal microscopy that clathrin colocalised with tubulin at mitotic spindles. Using a propidium iodide flow cytometric assay we found no statistical difference in the cell cycle distribution of the knockout cells versus the wild-type. Additionally, we showed that the ploidy and the recovery kinetics following cell cycle arrest with nocodazole were unchanged by repressing clathrin heavy chain expression.

**Conclusions/Significance:**

We conclude that the association of clathrin with the mitotic spindle and the contribution of clathrin to endocytosis are evolutionarily conserved. However we find that the contribution of clathrin to mitosis is less robust and dependent on cellular context. In other cell-lines silencing RNA has been used by others to knockdown clathrin expression resulting in an increase in the mitotic index of the cells. We show an effect on the G2/M phase population of clathrin knockdown in HEK293 cells but show that repressing clathrin expression in the DKO-R cell-line has no effect on the size of this population. Consequently this work highlights the need for a more detailed molecular understanding of the recruitment and function of clathrin at the spindle, since the localisation but not the impact of clathrin on mitosis appears to be robust in plants, mammalian and chicken B-cells.

## Introduction

Clathrin is a three-legged molecule with a central hub domain from which three ∼190 kDa heavy chains are extended, each ending in an N-terminal seven-bladed β-propeller domain that allows for multiple protein interactions with various specificities between its blades [1]. A single clathrin heavy chain (CHC) molecule contains in addition eight CHC repeat segments, a proximal hairpin, a tripod region believed to be responsible for trimerisation, and a variable C-terminal segment [2]. Each CHC is furthermore associated with a ∼25 kDa clathrin light chain (CLC). This building block of a cage structure is known as a triskelion, and during endocytosis the legs of neighbouring triskelia twist around each other to form a curved lattice that self-polymerizes around invaginated pits, stabilizing them as they bud from the major sites of formation within the cell–plasma membrane, trans-Golgi network and endosomes [3]–[5].

Recently, research has focussed on changes in the rates of endocytosis during cell cycle progression and in the distribution of trafficking proteins. This has resulted in some controversy in the literature over whether endocytosis is inhibited during mitosis or is maintained. An original study showed that in a broken assay mitotic cytosol could inhibit endocytosis when compared to interphase material [6]. Latterly, single-cell imaging has been used to determine that whilst endocytosis is maintained during all phases of the cell cycle, recycling of internalised membrane is inhibited during mitosis [7], [8]. Clathrin has also been found at the mitotic spindle both through confocal imaging and proteomic analysis of enriched spindle fractions [9], [10]. Knockdown of the heavy chain of clathrin in HEK293 and NRK cells using siRNA results in mitotic defects and this has led to the suggestion that clathrin may have a trafficking-independent function in mitosis [11]. By contrast, components of the AP-1, AP-2 and AP-3 adaptor complexes did not colocalise with the spindle apparatus [11]. Past controversy on changes in the endocytic rates during cell cycle progression suggests that it will prove important to explore the role of clathrin at the spindle in multiple cell-lines using multiple approaches.

Consequently, we have used a chicken pre-B lymphoma cell line DT40, which was generated with endogenous alleles for CHC replaced by human CHC under the control of a tetracycline-regulatable promoter, in order to investigate the role of clathrin in the evolutionarily conserved process of clathrin-mediated endocytosis [12], [13]. Following repression of clathrin expression, receptor-mediated and fluid-phase endocytosis were significantly inhibited in a surviving sub-line (DKO-R) [12]. We have now used this well-characterised model of membrane trafficking to quantitatively test for the first time, using flow cytometry, the impact of clathrin knockout on cell cycle progression in a suspension cell-line. We found no difference in the cell cycle distribution of the knockout cells versus the wild-type. Additionally, we observed that the ploidy and recovery kinetics following cell cycle arrest with the microtubule-depolymerising agent nocodazole were unchanged by knocking out clathrin. Consequently, whilst clathrin is an important component of the trafficking machinery and colocalises with the mitotic spindle, in these cells, its contribution to mitosis is not appreciable.

## Results

**Figure 1:**
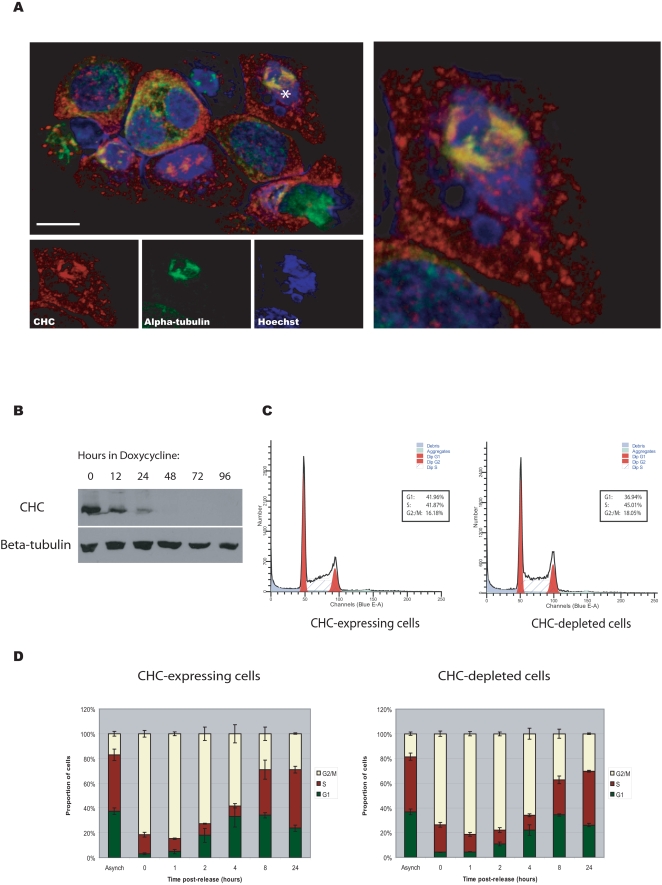
A) Clathrin heavy chain colocalizes with alpha-tubulin at the spindle apparatus in mitotic DKO-R cells. Confocal immunofluorescence microscopy on an asynchronised population of DKO-R cells grown in the absence of doxycycline (*upper left panel)*. Cells were stained with Hoechst 33258 (blue; Sigma), anti-alpha-tubulin DM1A (green; AlexaFluor 488 donkey anti-mouse conjugate, Invitrogen) and anti-CHC Ab21679 (red; AlexaFluor 594 goat anti-rabbit conjugate, Invitrogen). The asterisk indicates a mitotic cell in which CHC is targeted to the kinetochore fibres. A Z-stack projection of the indicated mitotic cell is shown (*right panel)*. Scale bar, 10 µm. B) Repression of CHC to undetectable levels is achieved after 48 hours treatment with doxycycline. Western blot analysis of DKO-R cells treated with doxycycline for the indicated time periods. β-tubulin was used as a loading control. C) Cell cycle distribution is unaffected by CHC depletion. DKO-R cells were treated with doxycycline for 48–72 hours and then subjected to flow cytometric analysis. DNA content was monitored by PI staining. The cell cycle profiles of CHC-expressing and CHC-depleted cells are shown. The percentage of cells in each cell cycle phase was calculated by ModFit Sofware and is shown in the boxed legend. D) The recovery kinetics from cell cycle arrest at the G2/M checkpoint are unaffected by CHC depletion in DKO-R cells. Time-course analysis of DKO-R cells following release from a nocodazole-induced block. Cells were grown in the presence (CHC-depleted) or absence (CHC-expressing) of doxycycline for 48–72 hours and then synchronized at metaphase by treatment with 500 ng/ml of nocodazole for 11 hours. A representative vehicle-treated asynchronous sample is indicated for each population (‘Asynch’ bar). At the indicated time points post-release from nocodazole block, cells were harvested and subjected to propidium iodide flow cytometric analysis. Data represent the mean values from three independent experiments with error bars denoting standard errors. The post-arrest recovery kinetics were compared for the clathrin-expressing and clathrin-depleted cells at each phase in the cell cycle using the analysis of variance (ANOVA). A p-value of 0.05 or less would be required to declare a significant difference between the two populations. P-values were 0.20 for G1-phase, 0.16 for S-phase and 0.09 for G2/M phase, indicating that there is no evidence of a difference between the recovery kinetics of the two populations over time following a nocodazole-induced block.

**Figure 2:**
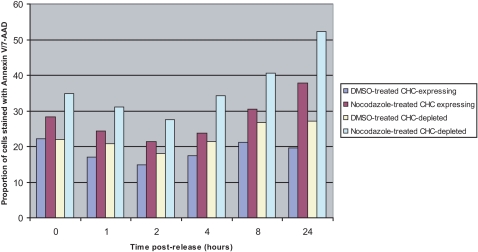
Time course analysis of cell death levels after release from a nocodazole-induced metaphase block. Asynchronous and nocodazole-arrested CHC-expressing and -depleted DKO-R cells were harvested at the indicated time points and stained with Annexin V and 7-aminoactinomycinD (7-AAD) followed by flow cytometry analysis. Although there is an increase in the levels of cell death during the recovery period, which has been well-established to be an effect of microtubule-targeting agents [25], there are no significant difference in the proportion of dead cells between the two populations. Data represents values from one experiment.

**Figure 3:**
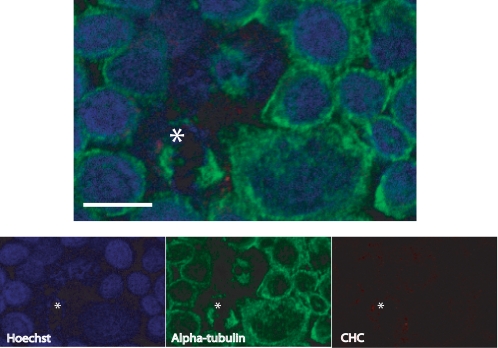
CHC-depleted DKO-R cells preserve the ability to assemble a mitotic spindle. Confocal immunofluorescence microscopy on an asynchronised population of DKO-R cells grown in the presence of 50 ng/ml doxycycline for 72 hours. Cells were stained with Hoechst 33258 (blue; Sigma), anti-alpha-tubulin DM1A (green; AlexaFluor 488 donkey anti-mouse conjugate, Invitrogen) and anti-CHC Ab21679 (red; AlexaFluor 594 goat anti-rabbit conjugate, Invitrogen). The asterisk indicates a mitotic cell. Scale bar, 10 µm.

**Figure 4:**
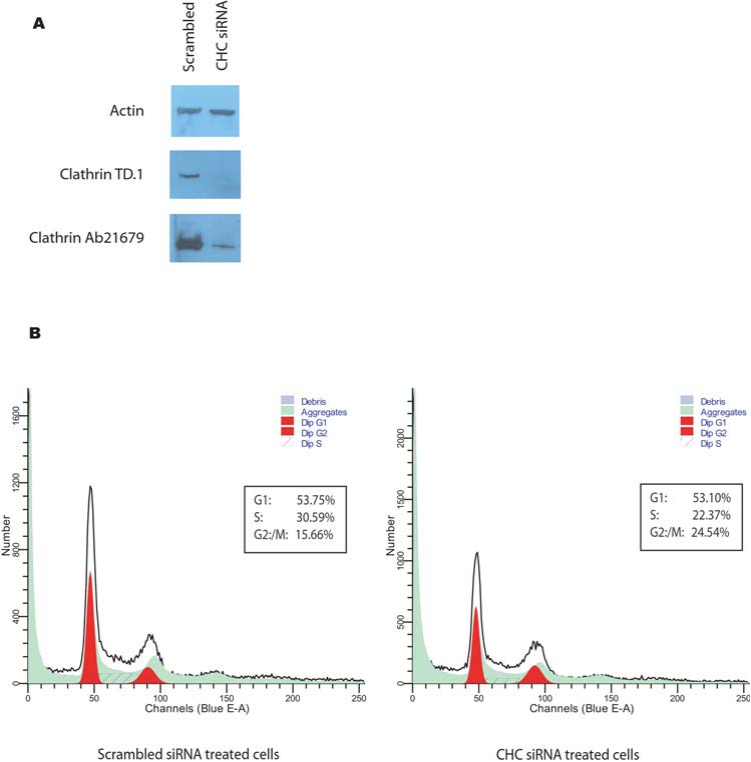
Clathrin knockdown in HEK293 cells leads to an increase in the G2/M phase content. A) Western blot analysis of HEK293 cells transfected with siRNA against CHC17 or with scrambled siRNA. β-actin was used as a loading control and two different clathrin heavy chain antibodies raised against the N-terminal domain (TD.1; Abcam) and the C-terminus (Ab21679; Abcam) were used on cell lysates harvested at the time of cell cycle analysis. B) The cell cycle profiles of scrambled siRNA treated and CHC siRNA treated cells are shown. The percentage of cells in each cell cycle phase was calculated by ModFit Sofware and is shown in the boxed legend.

We first monitored the spatial distribution of CHC and alpha-tubulin during cell cycle using confocal microscopy, confirming their colocalization at the spindle apparatus during mitosis in the DKO-R line (**Figure 1A**). We then treated the line with doxycycline (50 ng/ml) to fully repress CHC expression, which was achieved after 48 hours (**Figure 1B**). Royle *et al.* had shown an increase in the mitotic index of cells treated with siRNA against CHC using confocal microscopy. To test this more quantitatively, we sought to analyse steady-state cell cycle distribution in the presence and absence of CHC using a propidium iodide flow cytometric assay. Intriguingly, we found no change in the G2/M phase proportion of CHC-depleted cells relative to wild-type, or evidence of changes in their ploidy status (**Figure 1C**). Nevertheless, the absence of steady-state changes would not preclude differences in the transition rate through cell cycle checkpoints. To investigate this, we added nocodazole (500 ng/ml) for 11 hours to induce full spindle disruption and thus arrest clathrin-expressing and knockout cells at the G2/M checkpoint[14]. We then sampled the cells over a time course (1, 2, 4, 8 and 24 hours) having washed out the drug, and measured the proportions in each phase of the cell cycle by flow cytometry. There were no statistically significant differences in the recovery rates between clathrin depleted and control cells (**Figure 1D**). Furthermore, an apoptosis assay conducted on these cells over the same time course showed no significant differences in the level of cell death between the recovering populations (**Figure 2**). Finally, we confirmed that the clathrin-depleted cells are able to assemble mitotic spindles and are mononuclear (**Figure 3**). On the other hand, the same flow cytometric approach combined with targeting clathrin for knockdown using siRNA in a HEK293 cell-line resulted in an increase in the G2/M population from 15.66% to 24.54% despite the presence of detectable residual clathrin (∼10%) in total cell lysates, as detected using the same clathrin antibody (**Figure 4**). Consequently whilst the repression of clathrin in DKO-R cells produced a greater reduction in detectable clathrin than siRNA on HEK293 cells, the functional effects of this reduction were only measurable in HEK293 cells. This implies a difference in the mitotic sensitivity of the two cell-lines to changes in clathrin levels.

## Discussion

Given that clathrin does not seem critical for the function of the mitotic spindle in the DKO-R line, an alternative explanation for its localisation at the spindle apparatus remains that clathrin redistribution to the spindle is a by-product of Golgi disassembly which occurs during mitosis. Clathrin has been implicated in a number of nonendocytic functions including the maintenance of basolateral polarity involving the regulation of protein exit from the Golgi [15], and post-mitotic Golgi reassembly [16]. A number of proteins required for the maintenance of the Golgi stack are known to associate with the spindle [17] and clathrin redistribution may be a marker for their recruitment. Clathrin is a stable protein with a slow turnover [18] and consequently degrading and resynthesising this protein within the timeframe of M phase is not a feasible means of disassembling and regenerating subcellular organelles and vesicles during cell cycle progression. Our work strongly suggests that cell type and context are important determinants of the significance of the contribution that clathrin makes to mitosis, as has long been known for the role that it plays in endocytosis. For example, B cell antigen receptor internalisation was shown to be inhibited 70% by clathrin depletion in another DT40 variant cell-line (DKO-S), but to be unimpaired by depletion in the DKO-R line [19]. Clathrin has also been shown to contribute to the delivery of Lamp1 and lysosomal proteins to the lysosome through the application of siRNA to knockdown expression in HeLa cells [20]. By contrast, no lysosomal defects or abnormalities in the distribution of lysosomal proteins have been observed upon clathrin depletion in the DKO-R cell-line [12]. DKO-R cells are not normally grown in the absence of clathrin, there is merely inducible depletion. However, there remains the possibility of transient adaptive changes to clathrin depletion in these cells over the 72-hour timecourse used in the DKO-R experiments,as there might be in other cells treated with siRNA for up to 72 hours. An expression array study is currently underway to address this point further. Nonetheless, just as in other cell-lines, aspects of receptor-mediated and fluid-phase endocytosis are impaired by clathrin depletion in the DKO-R cell-line.

There is additionally a second clathrin heavy chain variant, CHC22, which is predominantly expressed in muscle and is highly similar in sequence to the ubiquitous CHC17 form introduced into the DKO-R cell-line. Whilst there is also a small amount of CHC22 detectable in this cell-line, it is not upregulated in response to doxycycline treatment and small levels are also found in HEK293 and HeLa cells (Dr. A. P. Jackson; Personal communication). Furthermore, CHC22 has not been detected at the mitotic spindle (Professor Frances Brodsky, UCSF; Personal communication). Consequently, we do not feel that compensation or adaptation to clathrin depletion through CHC22 is likely to happen in this case.

In conclusion, further detailed study is required to better understand at the molecular level the recruitment of clathrin to the spindle, as this represents a common feature of chicken B-cells, mammalian [10] and plant cells [21], and yet not of apparently equivalent functional significance for mitosis. Just as clathrin-independent or compensating endocytic mechanisms have been characterised, perhaps in the future both clathrin-dependent and clathrin-independent (or compensating) mechanisms for spindle assembly and chromosome segregation will be unearthed.

## Materials and Methods

### Cell Culture

DKO-R cells were cultured in RPMI media (Invitrogen) supplemented with 10% Foetal Bovine Serum (FBS; Invitrogen), 1% chicken serum (Invitrogen) and 10^−5^ M β-mercaptoethanol (Sigma) at 40°C in a humidified incubator supplied with 5% CO_2_ [22]. HEK293 cells were cultured in Dulbecco Modified Eagle's Medium (DMEM) containing 10% FBS at 37°C in a humidified incubator supplied with 5% CO_2_.

### Antibodies

All of the antibodies used in this study were obtained from Abcam (Cambridge, UK). Three clathrin antibodies were used; TD.1 (N-terminal domain epitope; Ab24578), X22 (C-terminal domain epitope; Ab2731), and Ab21679 (C-terminal epitope). Loading control antibodies were against beta-tubulin (D66; Ab11307) and actin (AC-40; Ab11003). In addition an alpha-tubulin antibody (DM1A; T9026) obtained from Sigma was used to stain microtubules.

### siRNA knockdown of clathrin in HEK293 cells

The procedure used has been adapted from Motley et al., [23]. Briefly HEK293 cells were seeded in full-serum medium into 100 mm dishes, at a density of 3×10^6^ cells per dish. The first transfection was carried out when cells had reached 80% confluency. For each 100 mm dish, 39 µl of DMRIE-C transfection reagent (Invitrogen) were added to 6 ml of serum-free DMEM, to which 70 nM of a set of 4 siRNA CLTC Duplexes (Dharmacon ON-TARGETplus Set of 4, Catalog No. LQ-004001-01) were added. Control siRNA was supplied by Qiagen (AllStars Negative Control siRNA (5 nmol Catalog No. 1027280). The transfection mixture was added to the cells after rinsing them once with serum-free DMEM, and the cells were left in this mixture until they were trypsinized the following day into two 100 mm dishes. 24 hours later a second transfection was carried out in a similar manner, and cells were equally trypsinized the following day and plated onto 100 mm dishes for protein extraction and cell cycle analysis 24 hours later.

### Western Blotting

Total cell extracts were prepared by lysing cells in Lysis Buffer containing 50 mM Tris-HCl pH 7.8, 150 mM NaCl, 5 mM EDTA, 15 mM MgCl_2_, 1% IgaPal, 0.75% sodium deoxycholate, 1 mM DTT, complete protease and phosphatase inhibitors (Roche) and 1 mM NEM. The extracts were cleared by centrifugation for 10 min at 14,200 rpm, followed by collection of the supernatant. Protein quantification was performed using Coomassie reagent and a BSA serial dilution for standard curve calculation. Cell lysates containing 15 µg of protein were analysed by 8% SDS-PAGE. Transfer to nitrocellulose membranes was carried out using the i-blot Dry Transfer System (Invitrogen), and membranes were immunoblotted with primary antibodies against clathrin heavy chain and beta-tubulin, followed by the appropriate HRP-conjugated secondary antibodies (Dako). Immunorreactive signals were visualized by ECL Plus (GE Healthcare).

### Apoptosis detection

DMSO- (vehicle) or nocodazole-treated wild-type and CHC-depleted DKO-R cells were stained with Annexin V-FITC (Cat# TA5532, R&D Systems, Inc.) and 7-actinomycinD (7-AAD; Invitrogen) according to the manufacturer's instructions, and subsequently analysed by flow cytometry on a four laser LSR II SORP flow cytometer (BD Biosciences, San Jose, CA). The fluorescence emitted by Annexin V-FITC and 7-AAD was collected using a 530/30 and 575/26 bandpass filter. Data collected from the experiment was analysed with FlowJo. V 8.3 (Treestar, Ashland, OR).

### Immunofluorescence and Confocal Microscopy

DKO-R cells were prepared for imaging as described previously[12] with the following exceptions. Coverslips were mounted using ProLong Gold antifade reagent (Invitrogen) with 1 µg/ml Hoechst 33258 (Sigma), and the following primary antibodies were used: anti-alpha-tubulin (DM1A, Sigma) and anti-CHC (ab21679, Abcam). Images were acquired on a Nikon Eclipse C1si confocal microscope system (Nikon UK Limited, Surrey, UK), and processed using Volocity image analysis software (Improvision, Coventry, UK).

### Flow Cytometric Analysis

DMSO- (vehicle) or nocodazole-treated wild-type and CHC-depleted DKO-R cells were stained with Propidium Iodide (Sigma) as described elsewhere[24], followed by analysis on a four laser LSR II SORP flow cytometer (BD Biosciences, San Jose, CA). The fluorescence emitted by the Propidium Iodide was collected using a 575/26 bandpass filter. Data collected from the experiment was analysed with ModFit LT V3.0 (Verity Software House, Topsham, ME).
